# The Relation Between Cardiac Output and Cerebral Blood Flow in ME/CFS Patients with a POTS Response During a Tilt Test

**DOI:** 10.3390/jcm14113648

**Published:** 2025-05-22

**Authors:** C. (Linda) M. C. van Campen, Frans C. Visser

**Affiliations:** Stichting CardioZorg, Kraayveld 5, 1171 JE Badhoevedorp, The Netherlands

**Keywords:** stroke volume, cardiac output, cerebral blood flow, tilt table test, orthostatic intolerance, chronic fatigue syndrome (CFS), myalgic encephalomyelitis (ME), healthy controls

## Abstract

**Background/Objectives**: Orthostatic intolerance is prevalent in patients with myalgic encephalomyelitis/chronic fatigue syndrome (ME/CFS) and is caused by an abnormal reduction in cerebral blood flow (CBF). In healthy controls (HCs), CBF is regulated complexly, and cardiac output (CO) is an important determinant of CBF. A review in HC showed that a 30% reduction in CO results in a 10% reduction in CBF. In contrast, we showed in ME/CFS patients with a normal HR (HR) and blood pressure response during a tilt test that CO and CBF decreased to a similar extent. The relation between CO and CBF in ME/CFS patients with postural orthostatic tachycardia syndrome (POTS) is unknown. Therefore, the aim of this study is to assess the relation between CBF and CO, in ME/CFS patients with POTS. The methods used in this retrospective study analyze this relation in a large group of patients. We also analyzed the influence of clinical data. A total of 260 ME/CFS patients with POTS underwent tilt testing with measurements of HR, BP, CBF, CO, and end-tidal PCO_2_. We measured CBF using extracranial Doppler flow velocity and vessel diameters obtained with a General Electric echo system, and suprasternal aortic flow velocities were measured using the same device. We recorded end-tidal PCO_2_ using a Nonin Lifesense device. **Results**: End-tilt HR and the HR increase were significantly higher in the patients with a %CO reduction ≥ −15% than in the other group. End-tilt CO was higher and the %CO reduction was lower in patients with %CO reduction ≥ −15% than in the other group. CBF data (supine, end-tilt and the %CBF reduction) were not different between the two patient groups. The use of HR increases and %SV reductions were not as discriminative as the %CO reduction. **Conclusions**: In ME/CFS patients with POTS during tilt testing with measurements of both the CO and the CBF, two different patterns were observed: (1) appr. two-thirds of patients had an almost 1:1 relation between the %CBF reduction and the %CO reduction. (2) Appr. one-third of patients showed a limited reduction in CO together with a substantial increase in HR. In these patients, there was no relation between the CO and CBF reduction. These data suggest the presence of a hyperadrenergic response.

## 1. Introduction

Regulation of cerebral blood flow (CBF) is complex, only partially understood and has been studied in healthy controls and patients with a variety of diseases. Mechanisms involved include the cerebral perfusion pressure [1], PO_2_ and PCO_2_ [2,3], flow-metabolism coupling [4], innervation of cerebral vessels [5], and blood viscosity [6]. Also, age and gender influence CBF. Although intuitively supposed that there is a tight relation between cardiac output (CO) and CBF in healthy controls, this cause–effect relationship is still debated [7,8]. Meng et al. described the relation between changes in CO versus changes in CBF in healthy controls during a variety of CO lowering interventions [7]. The authors found that a reduction of 30% in CO resulted in a 10% reduction in CBF. On the other hand, Castle-Kirszbaum et al. found in their systematic review that the “current literature is insufficiently robust to confirm an independent relationship between cardiac output and cerebral blood flow, and further studies with improved methodology are required before therapeutic interventions can be based on cardio-cerebral coupling” [8]. The relation between CO and CBF in patients with ME/CFS has seen limited investigation. Moreover, additional mechanistic abnormalities may play a role in ME/CFS patients: an abnormally decreased venous return due to orthostatic stress [9], differences in blood volume [10], leg venous distensibility [11], deconditioning [12], sympathetic drive [13], the hemodynamic abnormality during a tilt test [14], chronotropic incompetence [15], neuroinflammation [16], autoimmunity of the nervous system [17], endothelial dysfunction [18], and microclots [19] may play a role.

We recently published the linear relation between CO and CBF in ME/CFS patients and in healthy controls, both with a normal tilt (normal HR [HR] and blood pressure [BP]). The results showed that in a large majority of ME/CFS patients, the relation between CO and CBF is abnormal and significantly different from that of healthy controls: in healthy controls, a 10% reduction in CO resulted in a 6% reduction in CBF. This limited reduction in CBF for a given CO reduction is related to compensatory vasodilation of the cerebral vessels. In contrast, in 91% of ME/CFS patients with a normal HR and BP tilt test a 10% reduction in CO resulted in a 9% reduction in CBF, which may be indicative of endothelial dysfunction, limiting compensatory cerebral vasodilation [20].

We also recently described the relation between HR increase and stroke volume decrease during a tilt test in healthy controls and ME/CFS patients. Healthy controls and patients with a normal HR and BP during the tilt showed a linear relation between HR increase and stroke volume index decrease: higher HR increases were related with larger stroke volume index decreases [21]. In contrast, in ME/CFS patients with postural orthostatic tachycardia syndrome (POTS) during the tilt, this inverse relation was not present over the whole range of HR increases: in POTS patients with a limited HR increase up to 40 bpm, the inverse relation was preserved, similar to that of healthy controls and patients with a normal tilt test. However, in POTS patients with an HR increase above 40 bpm, the higher HR increases were accompanied by smaller stroke volume decreases. This was supposed to be related to increased catecholamine levels, increasing both HR and increasing contractility and thus reducing the stroke volume reduction.

Given these two observations, we hypothesized that ME/CFS POTS patients with a moderate HR increase during a tilt test show a similar relation between cardiac output reduction and cerebral blood flow to as ME/CFS patients with a normal HR and BP response (being abnormal and almost 1:1). Second, we hypothesized that in POTS patients with a high HR increase, the cardiac output reduction is less than in POTS patients with a moderate HR increase, leading to a lesser reduction in cerebral blood flow. Thus, the aim of the study was to assess these hypotheses.

## 2. Materials and Methods

The medical records of all ME/CFS patients who visited the out-patient clinic of the Stichting CardioZorg from November 2012 to June 2023 and who underwent a tilt test were reviewed. Only patients showing POTS during the tilt test were included in the present study. The patient selection and procedures were described extensively in a manuscript in this journal [21]. In short, patients were selected for analysis when from the tilt test; both Doppler data of CO and CBF were available both in the supine position and in the upright phase of the test. Patients were advised, when possible, to taper off and to stop drugs influencing HR or BP one week before the date of the tilt testing. During the first visit, we determined whether participants satisfied the criteria for ME and for CFS [22,23], taking the exclusion criteria into account. No other illnesses were present that explained symptomatology. Disease severity in patients was scored according to the international consensus criteria (ICCs), with severity scored as mild, moderate, severe, and very severe [23]. Very severe patients (bedridden patients) were not studied here because they were not able to undergo a tilt test.

The study was conducted in accordance with the Declaration of Helsinki. All ME/CFS participants and healthy controls gave informed, written consent. The study was approved by the medical ethics committee of the Slotervaart Hospital, Amsterdam, P1736.

### 2.1. Tilt-Test Protocol

Measurements were performed as described previously [14,24]. Briefly, all participants were positioned for 20 min in a supine position before being tilted head-up to 70 degrees. Tilt duration was maximally 30 min, but tilt duration was shorter and determined by the patient’s wellbeing and symptomatology; with increasing symptoms patients, were tilted for a shorter period to avoid syncope and to avoid possible post-exertional malaise.

HRs, systolic, and diastolic BPs were continuously recorded by finger plethysmography [25,26], using either a Nexfin (Edwards Lifesciences Corporation (BMEYE), Irvine, CA, USA), or Finapress (Finapress Medical Systems, Enschede, The Netherlands) device. After the test, HRs and BPs were imported into an Excel spreadsheet. The changes in HR and BPs were classified according to the consensus statement [27,28,29]: normal HR and BP response (normal HR-BP response), classic orthostatic hypotension (cOH), delayed orthostatic hypotension (dOH), POTS, and (near)-syncope. We only analyzed in the present study patients with POTS, defined by a sustained HR increase ≥ 30 bpm during the tilt test without a significant BP decrease, and accompanied by orthostatic intolerance and palpitation complaints [30].

### 2.2. Extracranial Doppler: Cerebral Blood Flow Measurements

Measurements were performed as described previously [14,24]. Internal carotid artery and vertebral artery Doppler flow velocity frames, combined with B mode images, were acquired by one operator (FCV), using a Vivid-I system (General Electric [GE] Healthcare, Hoevelaken, The Netherlands) equipped with a 6–13 MHz linear transducer. Frames were recorded in the supine position, before the onset of the tilt period, and while upright once or twice. When in the upright position, two sets of cerebral flow acquisitions were available; only the first set was analyzed. Blood flow of the internal carotid and vertebral arteries was calculated offline by one investigator (CMCvC). Blood flow in each vessel was calculated from the mean blood flow velocities multiplied by the vessel cross-sectional area and expressed in mL/minute. Flow in the individual arteries was calculated in 3–6 cardiac cycles and data were averaged. Total cerebral blood flow was calculated by adding the flow of the four arteries. For the analysis of the cerebral blood flow reduction during the tilt, the cerebral blood flow during the tilt was expressed as %cerebral blood flow reduction = cerebral blood flow_end-tilt_/cerebral blood flow_supine_ − 1.

### 2.3. End-Tidal CO_2_ Pressure Measurements

As blood CO_2_ pressures have a powerful influence on cerebral flow, both during hyper- and hypocapnia [2], we also measured end-tidal CO_2_ pressures (in mmHg) with the Nonin Lifesense device. End-tilt pressures below 30 mmHg were considered abnormal [31].

### 2.4. Doppler Echocardiographic Measurements

Time velocity integral (VTI) frames were obtained in the resting supine position and during the tilt phase, immediately after the cerebral flow acquisitions. The aortic VTI was measured using a continuous-wave Doppler pencil probe connected to a Vivid I machine (GE, Hoevelaken, The Netherlands) with the transducer positioned in the suprasternal notch. A maximal Doppler signal was assumed to be the optimal flow alignment. At least 2 frames of 6 s were obtained, and recordings were stored digitally. The VTI was measured off-line by manual tracing of at least six cardiac cycles, using the GE EchoPac post-processing software (Version 6.12.2) by one operator (CMCvC). The outflow tract diameter was manually drawn just below the valve insertion in the parasternal long-axis view of a previously made echocardiogram and the cross-sectional area was calculated. As the outflow tract is not circular but ellipsoid, we used the data of Maes et al. [32], to correct for the overestimation by the circular shape of the ellipsoid ventricular outflow tract calculation. In their study, the overestimation of the outflow tract area, using circular calculation by transthoracic echocardiography, was 24.5%. Therefore, we reduced the outflow tract area by 25%. Stroke volume was calculated from the aortic VTI, multiplied by the corrected aortic valve area, and expressed in mL. stroke volume of the separate cycles averaged. CO was calculated by the following formula: stroke volume multiplied by HR; it was expressed in L/min. The %CO reduction was calculated using the formula %CO reduction = CO_end-tilt_/CO_supine_ − 1.

### 2.5. Statistical Analysis

Data were analyzed using the statistical package of IBM SPSS, version 29.0.00.0. All continuous data were tested for the normal distribution using visual inspection of the Q-Q plots and presented as the mean and standard deviation (SD) or as the median with the interquartile range (IQR) where appropriate. Nominal data were compared using the Chi-square test (gender and disease severity, 3 × 2 and 3 × 3 tables). Group differences were explored using Students *t*-test, or by the Mann–Whitney U test. Due to the substantial number of comparisons, to reduce type I errors, we choose a conservative *p*-value of <0.01 to be statistically significant.

## 3. Results

From our database, we selected ME/CFS patients who visited our clinic between November 2012 and June 2023, who fulfilled the criteria for both ME and CFS [22,23], and who had a tilt test because of the suspicion of orthostatic intolerance (n = 1320). In all patients, CBF measurements as well as suprasternal derived stroke volume were available. From this group, we selected patients with POTS (n = 323). Patients younger than 18 years were excluded leaving 310 patients. Also, patients with a BMI > 40 kg/m^2^ were excluded, leaving 307 patients. Patients with insufficient quality of CBF or stroke volume measurements were excluded, as well as patients with missing data for CBF and stroke volume, leaving 299 patients. Finally, thirty-nine patients using HR- and BP-lowering drugs or lung medication containing sympathomimetics were excluded. This left 260 patients to be analyzed.

**Figure 1:**
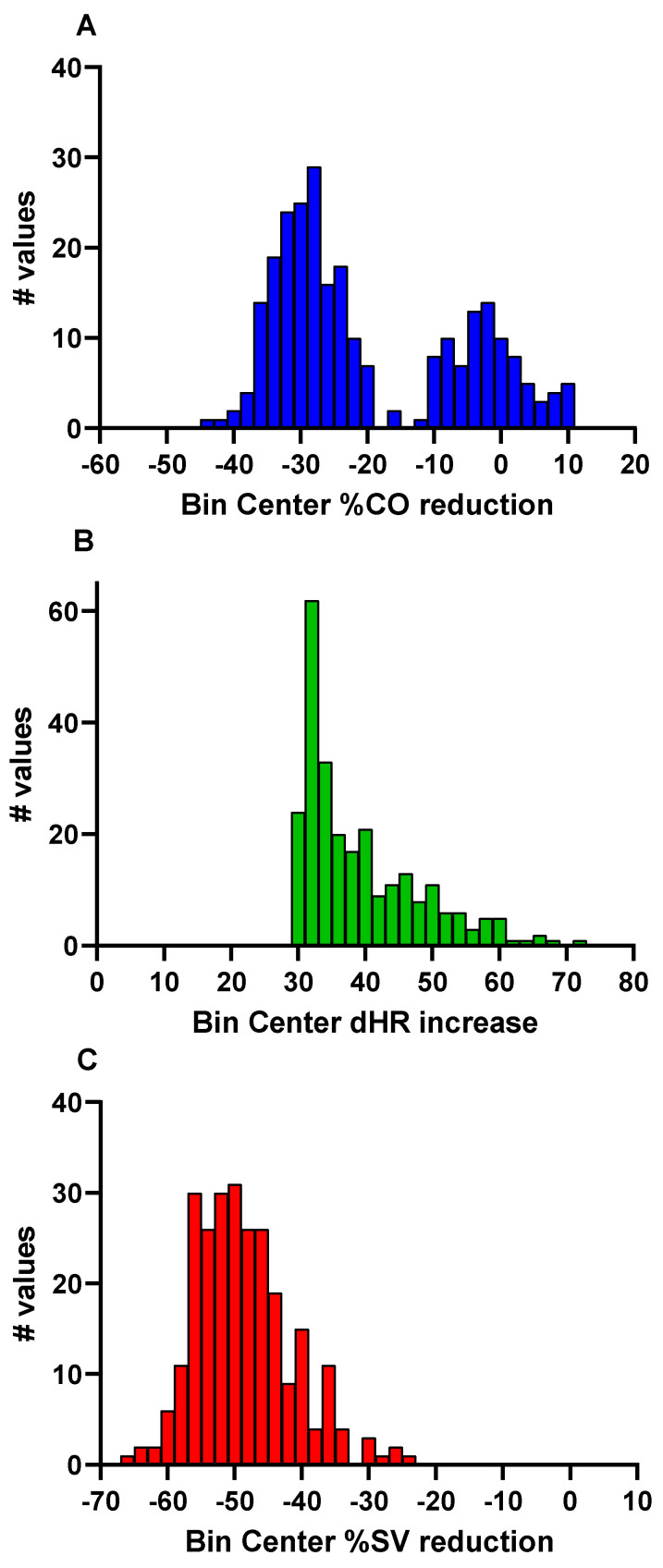
Histogram of the %CO reduction during the tilt test in ME/CFS patients with postural orthostatic tachycardia syndrome (POTS). %CO: percentage cardiac output reduction (**A**) during the tilt; dHR increase: HR increase (**B**) during the tilt; %SV reduction: percentage SV reduction (**C**) during the tilt; ME/CFS: myalgic encephalomyelitis/chronic fatigue syndrome.

Figure 1A–C show the histograms of the %CO reduction (Figure 1A), of the HR increase (Figure 1B) and of the %stroke volume reduction (Figure 1C) during the tilt. Two distinct groups of %CO reduction data were present; therefore, we decided to separate patients into two groups with a discriminating %CO cut-off value of −15%. The use of HR increases and %stroke volume reductions were not as discriminative as the %CO reduction. Table 1 shows baseline characteristics of the two groups. None of the baseline characteristics were significantly different between the two groups.

Table 2 shows the hemodynamic data of the two groups in the supine position and at the end of the tilt phase. End-tilt HR and the HR increase were significantly higher in the patients with a limited %CO reduction (≥−15%) than in the other group. End-tilt CO was higher and the %CO reduction was lower in patients with limited %CO reduction (≥−15%) than in the other group. CBF data (supine, end-tilt and the % CBF reduction) were not different between the two patient groups. End-tilt P_ET_CO_2_ was lower in the patients with a limited %CO reduction (≥−15%), and the P_ET_CO_2_ reduction was significantly larger than in the other group with a large %CO reduction (<−15%). Nevertheless, the percentage of patients with an P_ET_CO_2_ < 30 mmHg was not different between the two groups. BPs were not different between the two groups. Tilt duration was slightly but significantly shorter in patients with a limited %CO reduction (≥−15%) compared to the other patient group. Frame acquisition for CBF measurements lasted 3.2 (1.7) minutes without differences between the two groups. Acquisition of VTI measurements lasted 0.8 (0.3) minutes without differences between the two groups.

**Figure 2:**
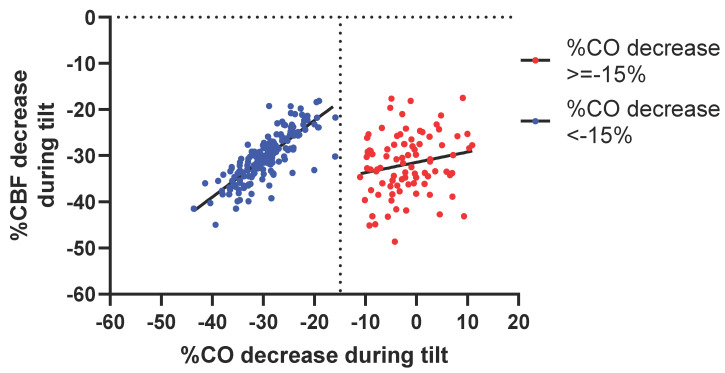
Relation between cardiac output reduction during the tilt and cerebral blood flow reduction during the tilt. CBF: cerebral blood flow; CO: cardiac output. The linear regression line of the blue dots (%CO decrease less than 15%) is Y = 0.8293X − 5.789; R^2^ = 80.33; *p* < 0.0001; for the red dots (%CO decrease more than 15%) it is Y = 0.2241X − 31.43; R^2^ = 19.06; *p* = 0.08.

Figure 2 shows the relation between the %CO reduction and %CBF reduction in the two groups. In patients with an abnormal and large %CO reduction (<−15%), the relation was highly significant: %CBF reduction = 0.8291 × %CO reduction − 5.789; R^2^ = 0.645; *p* < 0.001. In patients with a limited %CBF reduction (≥−15%), the slope was not significantly different from zero.

## 4. Discussion

In the present study, we have extended the analysis of a previous study on the relationship between HR increases and stroke volume decreases in ME/CFS patients with POTS. Here, we studied the CBF changes in relation to CO changes, these being the product of HR and stroke volume. The distribution analysis of the CO showed that there were two distinct groups: one with a limited decrease in CO during the tilt and one with a large decrease in CO < −15%. In the patient group with a limited CO decrease, the HR increase was significantly larger than in the patient group with a large CO decrease. Stroke volume reduction in the group with a limited CO decrease was significantly smaller than in the patient group with a large CO reduction. The P_ET_CO_2_ reduction was slight, but significantly larger in the patient group with a limited CO decrease compared to the patient group with a large CO reduction. Interestingly, despite major differences in CO reductions during the tilt between the two groups, the CBF reduction was not significantly different between the two groups. As in the two groups the CO reduction is significantly different and the CBF reductions are similar, the relations between the %CO reduction and %CBF reduction in the two groups are different: in patients with a large CO reduction, the relation was highly significant. In contrast, in patients with a limited CO reduction, the slope was not significantly different from zero.

In the present study, we have increased the number of patients with POTS (n = 260) [21] compared to our previous publication where 233 POTS patients were studied. The demographics and hemodynamic data of the added 27 POTS patients were like the previous POTS patient group.

The most important finding is that, based on the CO distribution, there are two distinctive POTS patient groups: those with a limited CO reduction during the tilt test and those with a large CO reduction during the test. In our previous publication, we showed that in patients with a limited HR increase, the HR–stroke volume relation followed that of ME/CFS patients with a normal HR and BP response: increasing HR values of individual patients were accompanied by a decrease in stroke volume [20]. In contrast, POTS patients with a high HR increase (above ± 40 bpm) showed a lower decrease in stroke volume reduction. This is hypothesized to be related to the effect of increasing circulating catecholamines which increase both HR and cardiac contractility. The increased contractility augment stroke volume, thereby decreasing the stroke volume reduction during the tilt in these patients. As CO is the product of HR and stroke volume, the CO reductions in both groups are different: in patients with a limited HR increase, the CO reduction is larger (due to a larger stroke volume reduction in combination with a smaller HR increase) than the CO reduction in patients with higher HR and lower stroke volume reduction during the tilt. Previous studies have shown that a subgroup of POTS patients have indeed a high level of catecholamines, mainly of norepinephrine [33,34,35], but also to a lesser degree of epinephrine [36]. This group of patients are commonly referred to as those with hyperadrenergic POTS. However, there is considerable overlap in norepinephrine concentrations between patients with and without a hyperadrenergic reaction [34]. Goldstein and Cheshire noted in their review that “drugs or disorders that inhibit Uptake-1 augment plasma norepinephrine for a given amount of norepinephrine release. This means that high plasma norepinephrine levels considered in isolation may not specifically indicate increased sympathetic nerve traffic” [37]. Moreover, when dividing the POTS patients into three different groups, being based on an underlying mechanism (hyperadrenergic, neuropathic and hypovolemic POTS), a considerable number of patients could be diagnosed with two or more underlying mechanisms, and importantly, that symptoms are similar across the phenotypes [34]. These considerations may limit the practical implications of phenotyping the various forms of POTS.

Between the two groups, there is also a difference in the relation between CO and CBF: in patients with a large CO reduction, there is a significant relation with the CBF reduction, being almost 1:1, while in patients with a limited CO reduction, there is no relation with the CBF: the slope is not different from zero. The almost 1:1 relation between CO reduction and CBF reduction was also present in ME/CFS patients with a normal HR and BP response during the tilt [20]. However, this relation is different from the CO-CBF relation in healthy controls: a 10% CO reduction resulted in a 6% CBF reduction using our extracranial Doppler technique [20] and a 3% CBF reduction using the transcranial Doppler technique [7]. It may be hypothesized that in these ME/CFS patients the compensatory vasodilation of the cerebral vessels during a significant CO reduction is almost absent and suggests endothelial dysfunction of cerebral vessels. Endothelial dysfunction in the brain has been described in a variety of cerebral diseases like Alzheimer disease [38], cerebral small vessel disease [39], multiple sclerosis [40], and possibly in Parkinson disease [41]. In ME/CFS patients, several studies have shown endothelial dysfunction using flow-mediated vasodilation/post-occlusive hyperemia [18,42,43,44]. In contrast, in POTS patients with a limited CO reduction, the CO-CBF relation is absent: this refutes our second hypothesis as mentioned in the introduction. The reasons for this absent relation are not clear, but it is evident from Figure 2 that inter-patient variability is larger in this group of patients than in the POTS patients with a large CO reduction. As stated above, we hypothesized that in the patient group with a limited CO reduction, elevated levels of catecholamines (hyperadrenergic response) are present. High levels of catecholamines in healthy controls result in an increase in CBF and oxygen consumption [45]. Others related the cognitive performance to dopamine and norepinephrine levels: this relation could be described as an inverted U shape where too high levels of catecholamines were related to a decrease in cognitive performance [46]. The decrease in cognitive function in the presence of high catecholamines may therefore lead to a decrease in CBF, called neurovascular coupling [47]. However, it does not explain the larger inter-patient variability. Possibly, there is a varying patient-related susceptibility for catecholamines. Thus, further studies relating the susceptibility, neuronal function, and CBF are needed.

Provided that the patient group with a limited %CO reduction comprises those with a hyperadrenergic response (which remains to be determined in further studies), the distinction between hyperadrenergic and non-hyperadrenergic reactions may be clinically relevant, as it is probable that those with a hyperadrenergic reaction may respond best on beta-blockers [48], while others may benefit from volume loading by increased water and salt intake, compression garments, fludrocortison, etc.; see Vernino et al. for an overview [49]. However, to date, there have been very few clinical interventional trials evaluating treatment for POTS and of treatment based on the various subgroups [50]. It is possible that the introduction of the CO during the tilt may help to differentiate the different forms of POTS and refine the treatment modalities. On the other hand, CO measurements are time-consuming and a costly procedure with an inherent learning curve. Finger plethysmography also presents data on stroke volume and cardiac output, but we previously demonstrated that this technique underestimates the changes during tilt testing [51]. The consensus meeting of the NIH identified multiple pathophysiological mechanisms leading to orthostatic intolerance and POTS [49]: hypovolemia, deconditioning, inflammatory mediators, excessive sympathetic stimulation, auto-antibodies, small-fiber neuropathy, and connective tissue laxity. However, other, rarely occurring mechanisms may also play a role, related to venous inflow obstruction: pericardial effusion due to viral pericarditis/myocarditis [52], effusion after COVID-19 vaccination [53], right atrial myxoma [54], constrictive pericarditis [55], and inflow obstruction due to pectus excavatum [56]. In summary, inflow obstruction leads to a reduced filling with a reduction in the end-diastolic volume and stroke volume. Improvement in diastolic filling results in an improvement of cardiac function and therefore a possible reduction in orthostatic intolerance, but this also depends on the disappearance/treatment of POTS [57]. There are limited data on the relation between the aforementioned mechanisms and the orthostatic intolerance, and this needs to be determined in detail in future studies.

In the present study, we found a small but significant difference in P_ET_CO_2_, this being two mmHg between the two groups. Although in healthy controls there is a powerful influence of the CO_2_ pressure on CBF [2], we previously found that the influence of CO_2_ on CBF during the tilt in a large group of ME/CFS patients with a normal HR and BP, or with POTS, is limited [58]. Therefore, this also applies to the present study.

Finally, we also found a small but significant difference in tilt duration, being on average a one min tilt duration difference. In a previous study with tilt duration differences of 12 min, the tilt duration did marginally contribute to the CBF reductions [20]. Therefore, the one-minute difference in the present study does not change the results.

### Limitations

The present study is limited in the following ways: firstly, it is retrospective; secondly, referral bias by the general practitioner may have played a role, selectively referring patients with orthostatic symptoms; although this might have affected the generalizability of our CBF measurements to the entire population of individuals with ME/CFS, it would not have interfered with the validity of the observed relationships between CO and CBF. The study did not include participants who were bedbound, and those with more severe functional impairments were not exposed to tilt testing. Furthermore, only patients with POTS during tilting were selected. The same analysis should be performed in patients with orthostatic hypotension. Individuals with ME/CFS have been reported to have variable function from day to day and week to week. Consequently, the potential differences in CBF and CO measurements on “good” versus “bad” days require further investigation. The present study’s primary focus was on the prevalence of reductions in CBF and CO, and therefore the mechanisms behind CBF and CO changes, and regional cerebral blood flow differences, were beyond the scope of this study. These topics merit further investigation. Finally, the use of extracranial Doppler flow to measure cerebral blood flow must be replicated by others and in different patient groups. The orthostatic intolerance exhibited by ME/CFS patients needs to be further delineated in comparison to other forms of circulatory dysfunction.

## 5. Conclusions

In ME/CFS patients with POTS during tilt testing with measurements of both the CO and the CBF, two different patterns were observed: 1) appr. two-thirds of patients had an almost 1:1 relation between the %CBF reduction and the %CO reduction. This CBF reduction is abnormal, as in healthy controls the CBF reduction for a given CO reduction varies between 1:3 and 2:3, and may indicate endothelial dysfunction with an inability of cerebral vessels to adequately dilate in the presence of a CO reduction. Appr. one-third of patients showed a limited reduction in CO together with a substantial increase in HR. In these patients, there was no relation between the CO and CBF reduction. These data suggest the presence of a hyperadrenergic response. If this mechanism is proven by norepinephrine levels, the CO is a robust measure to distinguish between hyperadrenergic and non-hyperadrenergic responses. This may lead to targeted therapeutic approaches being different between the two groups: circulatory improvement by increased water and salt intake, compression garments, and volume-expanding medication, as opposed to medication used to reduce the hyperadrenergic response (beta-blockers). This can be prospectively assessed in randomized, placebo-controlled trials.

## Figures and Tables

**Table 1:** Baseline characteristics of ME/CFS patients with postural orthostatic tachycardia syndrome (POTS) and with a limited %CO reduction (≥−15%) during a tilt test, or with a large %CO reduction (<−15%).

	ME/CFS and POTS with a Limited % CO Reduction (≥−15) (n = 88)	ME/CFS and POTS with a Large %CO Reduction (<−15%) (n = 172)	*p*-Value
Male/female *	7/81 (8/92%)	23/149 (41/59%)	0.196
Age (years) ∞	33 (10)	35 (10)	0.221
Height (cm) ∞	174 (7)	173 (8)	0.438
Weight (kg) #	64 (59–74)	68 (59–80)	0.146
BMI (kg/m^2^) #	21.4 (19.5–24.3)	24.2 (21.2–27.4)	0.393
BSA (m^2^)	1.77 (1.69–1.90)	1.81 (1.68–1.96)	0.048
Disease duration (years) #	10 (3–14)	9 (4–15)	0.865
Disease severity ^®^: mild/moderate/severe	21/40/27	38/92/42	0.431
	(24/46/31%)	(22/54/24%)	

BMI: body mass index; BSA: body surface area (formula duBois); ME/CFS: myalgic encephalomyelitis/chronic fatigue syndrome; POTS: postural orthostatic tachycardia syndrome; *: Chi-square analysis, ∞: median (IQR); #: Mann–Whitney U test; ^®^: disease severity according to the ICCs [23].

**Table 2:** Tilt test data of ME/CFS patients with postural orthostatic tachycardia syndrome (POTS) and with a %CO reduction ≥ −15% during a tilt test, or with a %CO reduction < −15%.

	ME/CFS with POTS and a Limited %CO Reduction (≥−15%) (n = 88)	ME/CFS with POTS and a Large %CO Reduction (<−15%) (n = 172)	*p*-Value
supine HR (bpm)	76 (13)	74 (12)	0.170
end-tilt HR (bpm) #	122 (114–132)	106 (98–114)	<0.001
HR increase (bpm)	49 (8)	34 (4)	< 0.001
supine SV (mL)	66 (12)	69 (12)	0.077
end-tilt SV (mL) #	37 (7)	33 (6)	<0.001
%SV reduction end-tilt	−43 (8)	−52 (5)	<0.001
supine CO (L/min) #	4.81 (4.34–5.47)	4.93 (4.22–5.56)	0.880
end-tilt CO (L/min) #	4.70 (4.29–5.32)	3.42 (3.00–3.97)	<0.001
%CO reduction end-tilt	−1.8 (5.6)	−29.4 (5.1)	<0.001
CBF supine (mL/min)	619 (94)	626 (106)	0.600
CBF end-tilt (mL/min)	410 (366–476)	428 (384–485)	0.131
%CBF reduction end-tilt	−31.8 (6.6)	−30.1 (5.2)	0.035
P_ET_CO_2_ supine	36 (4) n = 83	37 (3) n = 171	0.246
P_ET_CO_2_ end-tilt	25 (6) n = 83	27 (5) n = 171	0.001
P_ET_CO_2_ reduction (mmHg)	−11 (5) n = 83	−9 (4) n = 171	0.002
%pat P_ET_CO_2_ ≥ 30/<30 mmHg *	22/61 27/73%	68/103 40/60%	0.038
supine SBP (mmHg)	132 (15)	131 (15)	0.727
end-tilt SBP (mmHg)	127 (18)	127 (18)	0.948
supine DBP (mmHg)	79 (8)	80 (11)	0.454
end-tilt DBP (mmHg) #	86 (77–94)	87 (79–95)	0.436
MAP supine (mmHg)	100 (10)	100 (12)	0.801
MAP end-tilt (mmHg)	104 (13)	104 (16)	0.995
%MAP increase end-tilt	4 (10)	4 (13)	0.856
Tilt duration (min) #	7 (5–9)	8 (6–13)	<0.001

CBF: cerebral blood flow; CO: cardiac output; DBP: diastolic blood pressure; HR: HR; MAP: mean arterial pressure; ME/CFS: myalgic encephalomyelitis/chronic fatigue syndrome; P_ET_CO_2_: end-tidal CO_2_ pressure; SBP: systolic blood pressure; SV: stroke volume. * Chi Square test; #: Median (IQR) and Mann–Whitney U test; a *p*-value of <0.01 is considered statistically significant.

## Data Availability

No new data were created or analyzed in this study.
